# Preparation of acacia tannin loaded lipid microparticles by solid-in-oil-in-water and melt dispersion methods, their characterization and evaluation of their effect on ruminal gas production *In Vitro*

**DOI:** 10.1371/journal.pone.0206241

**Published:** 2018-10-25

**Authors:** Festus A. Adejoro, Abubeker Hassen, Mapitsi S. Thantsha

**Affiliations:** 1 Department of Animal and Wildlife Sciences, University of Pretoria, Pretoria, South Africa; 2 Department of Biochemistry, Genetics and Microbiology, University of Pretoria, Pretoria, South Africa; The University of Sydney, AUSTRALIA

## Abstract

Tannin extracts have wide biological activity in ruminant nutrition. The possibility of masking their bitter taste and enhancing sustained release in the rumen can be achieved through encapsulation. The objectives of this study were to prepare an encapsulated Acacia tannin extract (ATE) suitable for ruminants using the solid-in-oil-in-water (S/O/W) method, and to evaluate the microparticles in terms of morphology, encapsulation efficiency and *in vitro* release under varying pH. Subsequently, the effect of the microparticles on rumen *in vitro* gas and methane production would be evaluated. Lipid microparticles were prepared using the double emulsion process with palm oil and lard, dichloromethane, and Tween80/Span80 emulsifiers. The microparticles produced by S/O/W emulsion tended to be smaller (*P* = 0.06), and had greater encapsulation efficiency compared with those produced by the melt dispersion method. Scanning electron micrographs showed microparticles had stable cylindrical and spherical shapes, with mean size of 34± 10.2 μm. Maximum encapsulation efficiencies of 78.6% and 80.1% were obtained with lard and palm oil as lipid wall materials, respectively, even under high core material loading percentage of 80%. Wall material type did not affect the characteristics of microparticles. In acetate buffer, only about 20% of tannin was released from the lipid-encapsulated microparticles into buffer media after 24 hours. In contrast, about 90% of the tannin had been released into solution before eight hours in the crude extract. Lipid-encapsulated ATE reduced rumen gas and methane production *in vitro* (P ˂0.05) in both Eragrostis and total mixed ration (TMR) diet substrates, but the magnitude of reduction was lower than that obtained when unencapsulated ATE was the additive (10% vs 20% for total gas and 17% vs 24% for methane). Crude ATE and palm oil encapsulated ATE reduced the concentration of methane in sampled gas (*P* = 0.054) when fermenting the TMR substrate, but this effect was not observed in the Eragrostis substrate. Ammonia nitrogen concentration was greater in encapsulated ATE compared with the crude ATE (*P* ˂0.001). These results show that the lipid-encapsulated ATE produced small-sized and more uniform microparticles, with high encapsulation efficiency compared with microparticles prepared by melt dispersion. Encapsulation of ATE enhanced the sustained release of tannin in the rumen, and with the potential to improve gas production and reduce methane production.

## Introduction

Tannin extracts as dietary additives are gaining recognition in ruminant production because of their many nutritional, and nutraceutical uses. Their many biological functions include prevention of bloat, aiding the bypass of dietary protein, control of gastrointestinal nematodes and rumen methane mitigation [1–4]. However, the limitations to the use of tannins include stability, astringency and bitter taste, and its strong affinity with dietary ruminal protein, which at high concentration may result in reduced voluntary dry matter intake and nutrient digestibility among other negative consequences [5]. Recent studies have suggested that microencapsulation technique may be able to mask or reduce the negative effects associated with bioactive compounds in the food or feed industry [6]. Fang and Bhandari, [7] noted that the administration of encapsulated extracts instead of the raw product could overcome drawbacks such as product instability and bitter taste while improving the bioavailability of the compounds at the required site. A tannin extract that is release gradually over a prolonged period in the rumen may improve tannin utilization in ruminant nutrition [8].

Microencapsulation has been described as a process by which small particles of a core material are surrounded by a homogenous or heterogeneous coating (wall material) and form capsules or beads using various applications [9]. Active ingredients are encapsulated to achieve purposes such as masking unpleasant tastes or colours, extending shelf life, protecting against oxidative damage, and controlled release or release at a targeted site [10,11]. Controlled-release systems are specialized encapsulation systems, which deliver the active ingredients over an extended period [6]. These systems have been used in the administration of anthelmintics and antiparasitic drugs, antibiotics, vitamins, amino acids and other dietary additives for ruminants and humans [10]. They have shown important benefits by reducing the frequency of administration and minimizing negative side effects such as acute toxicity, rumen pH fluctuation, or astringency [12].

However, the cost, availability and suitability of many of the food industry encapsulating materials for animal nutrition have been highlighted [13,14]. This could hamper their commercial application in ruminant systems. Therefore, there is a need to develop and evaluate cheap but effective wall materials for various livestock applications. Multiple emulsion systems such as the S/O/W method offer innovative approaches to the administration of bioactive compounds such as anthocyanidins across the gastrointestinal passage because they are able to mask flavours and or odours or control the release of ingredients during ingestion and digestion [15,16]. Membrane emulsification methods such as S/O/W system could control particle size and enhance the stability of microparticles, unlike simpler techniques such as melt dispersion or solvent evaporation methods [17–19].

In this study, palm oil and lard (fat rendered from a pig) were used as lipid wall materials to encapsulate Acacia (*Acacia mearnsii*) tannin extract (ATE) in an S/O/W emulsion using Tween80 and Span80 as hydrophilic and lipophilic surfactants respectively. The lipid wall materials are economically viable, eco-friendly and can be hydrolysed and biohydrogenated in the rumen [20–22]. Nutritionally, there is an increase in fatty acid bioavailability to the animal [21]. Lard and palm oils have high melting points compared with many other vegetable oils, and, hence, microparticles prepared from them may be able to withstand disintegration caused by handling [23]. Lard has been used extensively as a milk replacer for feeding calves, and therefore does not impose health risks on ruminant animals [24].

The hypothesis of this study is that encapsulated ATE will result in microparticles that exhibit slower *in vitro* release, compared with the crude extract, while retaining its characteristic properties such as reduction in methane gas. The S/O/W double emulsion method was adopted based on its controlled-release advantages, and the microparticles were compared with microparticles prepared under melt dispersion, a simple method that has similarly been used for ruminant additives [25]. The objectives of this study, therefore, were i) to prepare an encapsulated ATE using the S/O/W method with lard or palm oil as wall material, then compare it with the product of the melt dispersion method, ii) to evaluate the microparticles in terms of morphology, efficiency of encapsulation, and the *in vitro* release profile of the ATE under varying pH conditions, and iii) to evaluate the addition of microparticles from ii in terms of their effect on *in vitro* gas and methane production as compared with the crude extract.

## Materials and method

This study was carried out in accordance with the guidelines stipulated by the National Health Research Ethics Council of South Africa. The protocol was approved by the University of Pretoria Animal Ethics Committee (AEC) (approval number EC061-14).

### Materials

*Acacia mearnsii* tannin extract (ATE) from UCL Tannin Pty (Ltd), South Africa, was used throughout this study. Span80 (HLB, 4.3), Tween80 (HLB, 15.0) and dichloromethane (99.9%, ACS HPLC grade) were procured from Sigma-Aldrich (Ltd) (USA). Filter bags used for *in vitro* release was the F57 fibre filter bags purchased from ANKOM Technology (NY, USA). All reagents were of analytical grade in purity.

### Microparticle preparation

Acacia tannin extract was a commercial sample obtained from UCL Tanning Company Pty (Ltd), Dalton, South Africa, and extracted from the bark of the black wattle (*Acacia mearnsii*) tree by steam distillation and then concentrated into powdered form. It has a molecular weight that ranges from 500 to 3000, with an average of 1250. It has a high amount of condensed tannin, although it also contains other non-tannins (including low molecular weight polyphenols, salts, sugars, and organic acids). From laboratory analysis, the sample had total phenol, total tannin and condensed tannin concentrations of 65.8%, 58.5% (as tannic acid equivalent) and 30.5% (as leucocyanidin equivalent), according to the procedure of Makkar et al. [26] and Porter et al. [27]. Dichloromethane, the solvent used in this preparation is the least toxic of the simple chlorohydrocarbons. The LD50 value is 1600 mg/kg via oral administration in rats and it is reported to be non-toxic to aquatic life [28]. Besides, it is commonly used in the food and pharmaceutical industries. The solvent was evaporated during microparticle preparation, and therefore its use was not likely to create a toxicity risk to animals.

**Fig 1:**
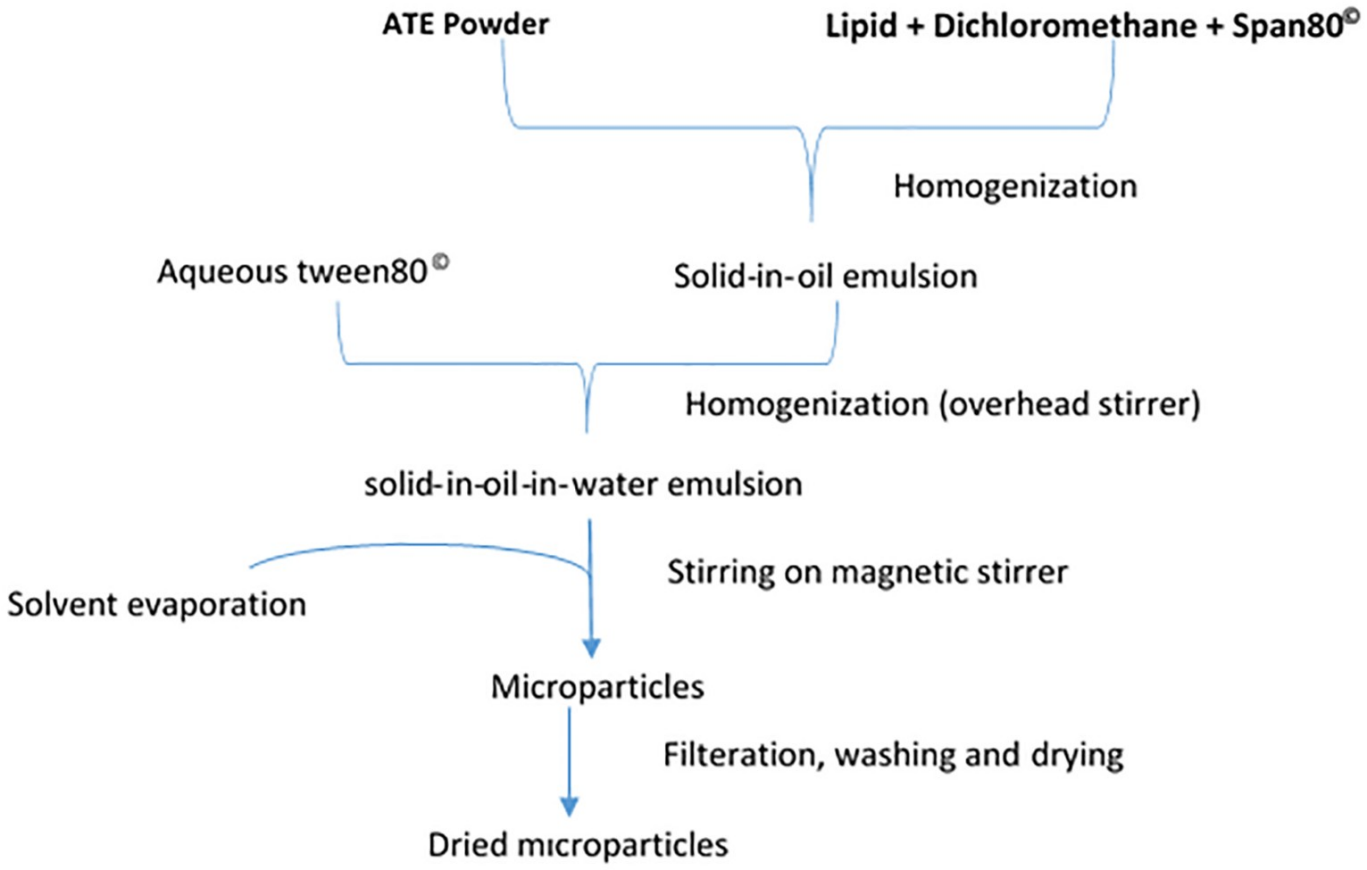
Schematic diagram showing the preparation of solid-in-oil-in-water microparticles.

Solid-in-oil-in-water method: The double-step procedure used in the preparation of multiple phase emulsions as described by Castellanos et al. [29] was used for encapsulation of ATE by the S/O/W method. The primary solid-in-oil (S/O) phase was prepared by suspending ATE powder in 30 mL lipid solution (50 mg/mL) of dichloromethane (DCM) containing Span80 as a surfactant and homogenized at 20,000 rpm for 120 seconds (PRO400DS, Pro Scientific Inc., Oxford CT 06478 USA). The resulting S/O suspension was added to an aqueous phase of distilled water containing 0.1% (w/v) Tween80 and homogenized for 180 seconds at 20,000 rpm to produce a secondary S/O/W emulsion. Varying concentrations of the ATE and external aqueous phase were compared to determine the optimum conditions for encapsulation. The resultant emulsion was placed on a magnetic stirrer and rotated at 800 rpm for three hours to allow for evaporation of the water-immiscible organic solvent (DCM). The microparticles that were formed were collected by filtration through a 60 μm glass filter crucible, washed with approximately 100 mL distilled water, freeze-dried and stored at 4°C for analysis. Preliminary tests were conducted to establish the best amount of Tween80 and Span80 combination that would give good emulsion stability. The parameters that were kept constant during the preparation of the S/O/W microparticles included concentration of surfactants (0.5% w/v Span80 in DCM and 0.1% w/v Tween80 in water), homogenization conditions (S/O phase: 20,000 rpm, 120 s and O/W phase: 20,000 rpm, 180 s) and solvent evaporation conditions (800 rpm, 3 h). The schematic chart of the encapsulation procedure is shown in Fig 1. The various preparation concentrations of the external aqueous phase and tannin extract are designated L-1 to L-4 in lard-encapsulated microparticles and P-1 to P-4 in palm-oil encapsulated microparticles.

Melt dispersion method: Preparation of the microparticles using the melt dispersion method was done according to the procedures of Richardson [30] and Wallace et al [31] with minor modifications. Briefly, 2 g palm oil or lard was added to a beaker and melted under gentle heat and continuous stirring using a magnetic stirrer until the liquid was clear. Acacia tannin extract powder (8 g) was placed in an IKA A10 electric mill and the melted lipid sprayed over the powder, and then the mixture was blended for two minutes. Thereafter, a spatula was used to scrape any powder that had stuck to the sides and lid of the mill, and the blending was repeated several times until the powder was thoroughly coated with the oil. Then, 120 mg of Span80 was added and the blending was repeated several times. Coated samples were passed through a six-mesh screen to break up agglomerates and then stored in an airtight container at 4°C. The melt dispersion method is considered simple and has been similarly used in ruminants [30,31]. It was therefore used to compare microparticles prepared by the S/O/W method.

### Microparticle morphology and size determination

Morphology of microparticles and particle size distribution were evaluated using scanning electron microscopy. The encapsulated powder was coated with carbon before sputtering with gold under an argon atmosphere (Emitech K550X, Ashford, UK), followed by viewing under a JSM-840 microscope (JEOL, Tokyo, Japan). The size of the microparticles was determined by comparing the scanning electron microscopy images with those of a scale bar of the same magnification. At least 50 microparticles of each treatment were measured.

### Determination of tannin yield and encapsulation efficiency of microparticles

The actual loaded tannin (L_A_) of microparticles was determined using a procedure described by Castellanos et al [29] with slight modifications. Briefly, 100 mg of microparticles were dispersed in 20 mL DCM, and the lipid wall material was dissolved by sonication for 10 minutes. The solid tannin was pelleted by centrifugation for 10 min at 2500 rpm. The supernatant was discarded and the pellet was dissolved in 20 mL 70% aq. acetone. The tannin concentration in the resulting clear solution was determined from its absorbance spectrophotometrically to estimate actual loaded tannin. The encapsulation efficiency of the microcapsules enclosing ATE was estimated as follows:

$$Ee(\%)=(\frac{L_{A}}{L_{T}})X\;100$$

where L_A_ is the actual loaded tannin and L_T_ is the theoretical loaded tannin (%, w/w) in the lipid microparticles. The theoretical loaded tannin was the actual weight of tannin added during preparation of the microparticles.

### *In vitro* release of tannin from microparticles

Many *in vitro* release studies use diffusion cells or dialysis tubes for the evaluation of the release of bioactive compounds from solid microparticles [32,33]. In this study, F57 ANKOM filter bags with porosity of 25 μm were used. These filter bags are designed for rumen fermentation simulations. The *in vitro* release of tannin extract from the encapsulation matrix in the gut of ruminant animals was simulated using product solubility in various pH media following the procedure of Rossi et al. [34]. Elution media used were acetate buffer (pH 5.4), phosphate buffer (pH 6.8) and HCl buffer (pH 2.2). Microparticles (100 mg) were weighed into cellulose filter bags with porosity of 25 μm (F57; ANKOM), suspended in 50 mL of elution media and then vortexed at 50 rpm at 39°C. Aliquots of 2 mL were removed at 30 min, and at 1, 2, 4, 8, and 24 hours after incubation. The initial volume was maintained by the addition of fresh buffer media. Aliquots were frozen immediately for subsequent analysis. The release of tannin was monitored by spectrophotometric evaluation of the samples as described above. The obtained release data for acetate buffer were applied in zero order, first order and Higuchi square root equations to find the best prediction of the release of tannin extract in the rumen, where tannin release is of nutritional interest [35].

### *In vitro* gas production

The *in vitro* gas production procedure detailed by Menke et al. [36] was used to evaluate the effect of lipid-encapsulated ATE on gas and methane production and on rumen fluid parameters. During the *in vitro* procedure, a semi-automated gas pressure transducer and digital tracker were used to record gas pressure at time intervals and converted to gas volume while gas samples were analysed for methane concentration using gas chromatography (8610C BTU Gas Analyser GC System, SRI Instruments, Germany). Rumen fluid was obtained from three Merino Rams, feeding *ad libitum* on Lucerne hay, mixed with buffer under continuous CO_2_ flushing, and used as inoculum (40 mL/bottle). Detailed procedures are described in Adejoro and Hassen [37]. *Eragrostis curvula* hay (CP, 55 g/kg; NDF, 784 g/kg; ADF, 492 g/kg) and a TMR sheep diet (CP, 180 g/kg; NDF, 301 g/kg; ADF, 214 g/kg) were used separately as substrates (400 mg DM). For each substrate, treatments include i) diet only, ii) diet plus crude ATE iii) diet plus lard-encapsulated ATE iv) diet plus palm oil-encapsulated ATE v) diet plus palm oil only vi) diet plus lard only. To each treatment containing ATE or encapsulated-ATE was added an equivalent of 30 mg ATE, which corresponds to 2.63% CT (leucocyanidin equivalent). Lard only and palm oil only treatments were added in amounts that were equivalent to those of the wall materials present in the lipid-encapsulated ATE to account for gas produced because of the wall material inclusion. Rumen fluid only incubation was included in each run to account for fermentation arising from the rumen fluid. The volume from this incubation was subtracted from gas volume for each time point. Four replicate bottles were incubated for each treatment with three repeated incubation runs. Gas production and methane concentration were measured at 2, 4, 8, 12 and 24 hours after incubation and cumulated to obtain the cumulative gas production at the time points. The rate and extent of gas production were determined by fitting the gas production data into the non-linear equation *y* = *a* + *b* (1 − *e*^−*ct*^) of Ørskov and McDonald [38] where, *y* = gas production at time *t*, a = gas production from the soluble fraction (ml g^-1^ DM), *b* = gas production from the insoluble but slowly fermentable fraction (mL g^-1^ DM), and *c* = rate of fermentation of fraction ‘*b*’ (mL h^-1^). Rumen fluid pH after 24 hours incubation was measured using a pH meter (Mettler Toledo 230 pH meter) while ammonia-nitrogen concentration was analysed as described by Broderick and Kang [39].

### Statistical analysis

For the *in vitro* gas production within each substrate, individual bottles for each treatment in each incubation run served as analytical replicates while each repeat incubation run served as a statistical replicate. Gas volume was plotted against incubation time, using the non-linear equation to predict fermentation kinetics variables [38]. Data on microparticle characteristics, gas production and fermentation parameters were expressed as least square means and were analysed using the PROC MIXED Procedure of SAS 9.4 (SAS Inst Inc, Cary, NC). The model statement included Yhijk = μ + Sh + Ri + Tj+ Qk + + SHTj + eij where, Y_hijk_ = mean of individual observation, μ = overall mean, S_h_ = effect of substrate, R_i_ = effect of incubation run, T_j_ = effect of treatment/additives, Q_k_ = effect of run within treatment, S_h_T_k_ = substrate-treatment interaction effect, and e_hijk_ = residual error. Incubation run and run within treatment were set as random effects, whereas substrate, treatment and substrate-treatment interaction were fixed effects. Mean separation was done using Tukey’s test.

## Results

### Characterization of microparticles

**Fig 2:**
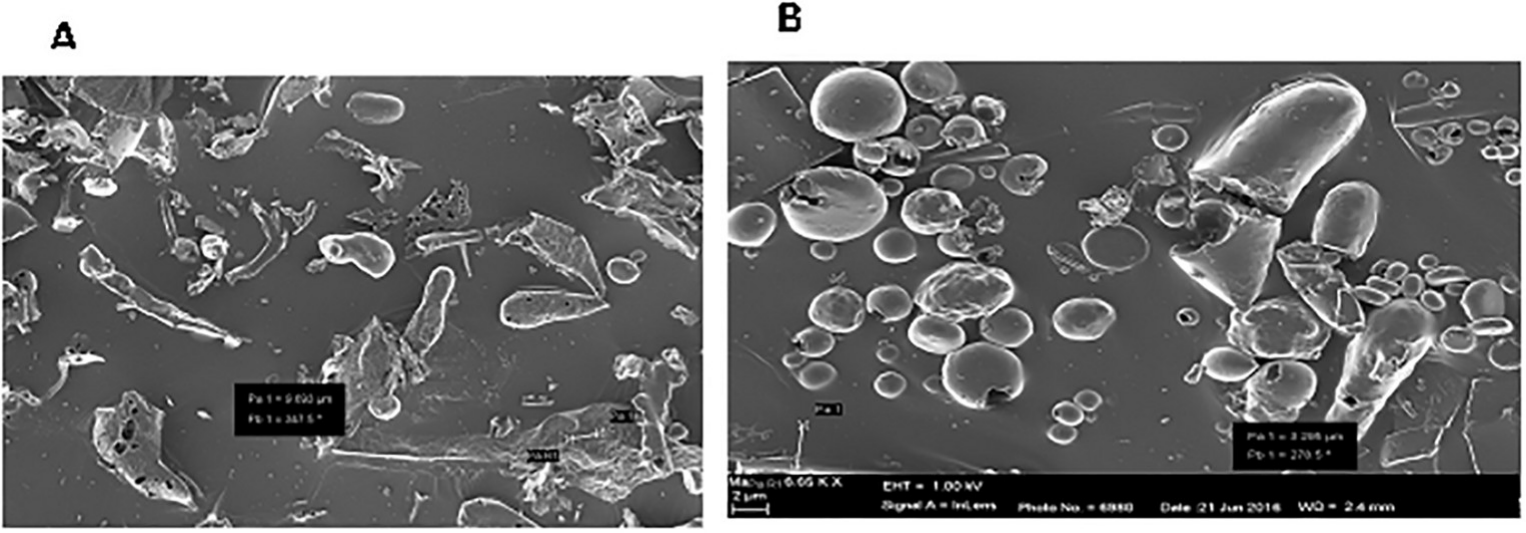
Scanning electron microscopy images of freeze-dried Acacia tannin extract lipid microparticles prepared by solid-in-oil-in-water encapsulation method using (a) lard and (b) palm oil.

The scanning electron micrograph images of ETE^L^ and ETE^P^ microparticles obtained under optimal conditions is shown in Fig 2. These microparticles had a mean diameter of approximately 34 μm and the encapsulation efficiency (EE) of approximately 80% (L-1; P-1) (Table 1). Encapsulation efficiency, yield and particle size in the S/O/W emulsion process with palm oil (ETE^P^) and lard (ETE^L^) compared with microparticles prepared by the melt dispersion method showed that core material concentration and volume of the external aqueous phase affected microparticle properties. Microparticles prepared by the melt dispersion technique resulted in a high yield of encapsulated tannin (95%) but low EE (46%) compared with microparticles prepared by the S/O/W emulsion method (*P*˂ 0.0001). The melt dispersion method produced microparticles that were more spherical in shape but were larger compared with the cylindrical microparticles obtained from the S/O/W emulsion process (*P* = 0.057). Based on EE and mean particle diameter, the lipid-encapsulated ATE microparticles from L-1 and P-1 were subsequently used in the *in vitro* release and *in vitro* gas production tests.

**Table 1:** Encapsulation efficiency and particle size of Acacia tannin extract-lipid microparticles prepared using melt dispersion and solid-in-oil-water encapsulation techniques.

			Yield (%)	Encapsulation efficiency (%)	Mean particle diameter (μm)
[A] Melt dispersion			94.5^a^	46.0^c^	58.0^a^
[B] Solid-in-oil-in-water					
Batch	ATE conc. (g)	External aqueous phase (mL)	Yield (%)	Encapsulation efficiency (%)	Mean particle diameter (μm)
L1	8.5	300	57.3^d^	78.6^a^	33.9^bc^
L2	9.0	300	65.7^c^	75.1^ab^	39.5^bc^
L3	8.5	500	71.4^bc^	68.6^b^	44.7^abc^
L4	9.0	500	73.2^b^	74.6^ab^	48.6^ab^
P1	8.5	300	63.1^cd^	80.1^a^	26.8^c^
P2	9.0	300	65.8^c^	77.8^a^	32.7^bc^
P3	8.5	500	72.5^b^	74.3^ab^	30.4^bc^
P4	9.0	500	74.5^b^	68.8^b^	36.5^bc^
SEM			2.03	2.05	2.55
P-value			˂0.0001	˂0.0001	0.057

^1^L: batches 1–4 of S/O/W microparticles prepared using lard as wall material; P: batches 1–4 of S/O/W microparticles prepared using palm oil as wall material. Mean values with different superscript within the same column are significantly different (*P* ˂ 0.05). Mean values are calculated from a minimum of three repeat batches.

### *In vitro* release behaviour of Acacia tannin extract encapsulated-lipid microparticles

**Fig 3:**
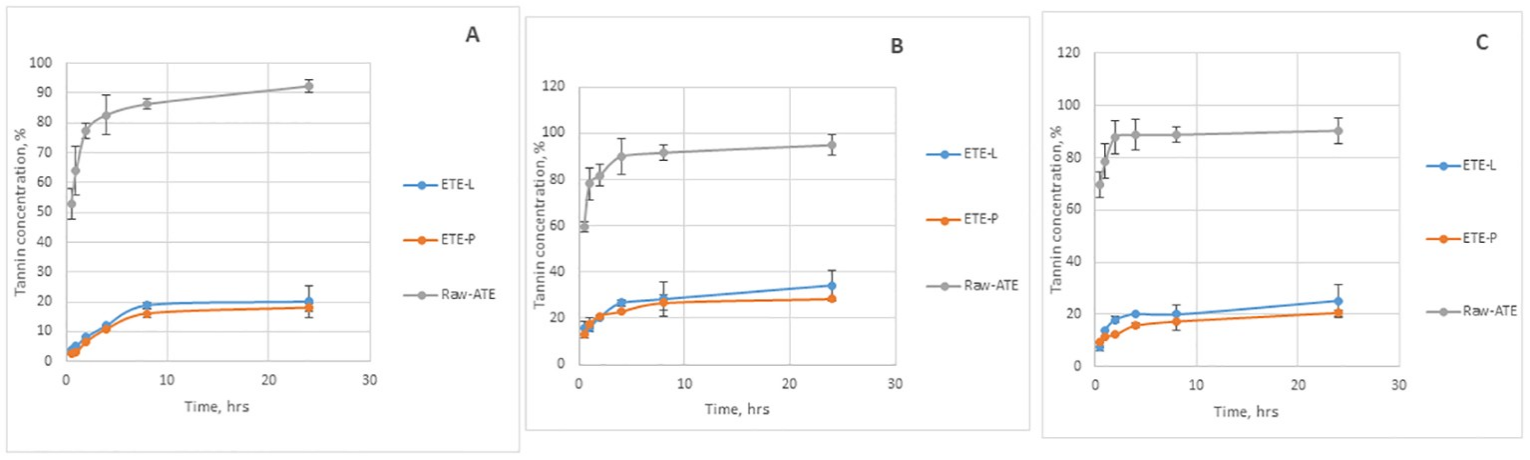
Release profiles of Acacia tannin extract and tannin extract microparticles encapsulated with lard and palm oil prepared by solid-in-oil-in-water encapsulation method in (A) acetate (0.1M, pH 5.5), (B) phosphate buffer (0. I M, pH 7.4) and (C) HCl buffer (0.1M, pH 2.2).

The percentages of ATE released in different dissolution media from the lipid microparticles with lard (ETE^L^) or palm oil (ETE^P^) as wall material are shown in Fig 3. The unencapsulated ATE produced a burst release in all dissolution media with 65% release within 2 hours and about 90% release before 8 hours of incubation. A slow release pattern, however, was obtained for the lipid-encapsulated ATE products. After 24 hours in the dissolution media, 20%, 34% and 25% of the extract was released from the ETE^L^ microparticles in acetate, phosphate and HCl media, respectively while 19%, 30% and 22% was released from the ETE^P^ microparticles in the same buffer media, respectively. The release of ATE from both lipid matrixes in acetate buffer followed the Higuchi equations better than the zero order and first-order equations (Table 2).

**Table 2:** *In vitro* release kinetic parameters in acetate buffer media (pH, 5.6), of Acacia tannin extract, or encapsulated Acacia tannin extract prepared with lard and palm oil using the solid-in-water-oil method (*n* = 3).

	Zero Order, Q vs. t	First Order, ln (Q0-Q) vs. t	Higuchi, Q vs √ t	R^2^		
				Zero Order	First order	Higuchi
ETE^L^	Y = 0.7988X+5.8823	Y = -0.0079X+1.9854	Y = 6.2174X-0.4151	0.6814	0.9177	0.9854
ETE^P^	Y = 0.7504X+4.6174	Y = -0.0072X+1.9899	Y = 5.6666X-1.0398	0.7042	0.9292	0.979
ATE	Y = 2.2159X+53.925	Y = -0.0658X+1.6814	Y = 21.702X+29.357	0.3537	0.7366	0.7082

Q0, tannin to be released at zero time (mg); Q, amount of tannin released at time t: time in hours

### *In vitro* fermentation, total gas production and methane emission of Eragrostis hay and a total mixed ration as influenced by lipid-encapsulated Acacia tannin extract

Table 3 shows *in vitro* gas production over a 24 hour period as affected by the incubation of the *E*. *curvula* (EC) and TMR substrates with the ATE-encapsulated lipid microparticles. There was a strong interaction effect between substrate type and treatment on the cumulative gas and methane production at each of the incubation times (*P*< 0.05) except methane production at 2 hours. Generally, regardless of the inclusion of additives, gas and methane volume was higher in the TMR substrate compared with the EC substrate across the incubation times. When both substrates were incubated with lipid wall materials only (lard or palm oil), there was no difference (*P*< 0.0001) in gas production at 24 hours, when compared to the diet only (controls). At 24 hours, incubation with crude ATE reduced the total gas production (mL/g DM) by 19.5% and 18.8% for EC and TMR substrates, respectively, compared with the controls. The ETE^L^ and ETE^P^ incubations reduced (*P*˂ 0.0001) 24 hour total gas production when expressed in mL g^-1^ DM by 6.8% and 7.2%, respectively in the EC substrate and by 13.4% and 13.2%, respectively, in the TMR substrate when compared with the controls (diet only). The inclusion of crude ATE reduced methane production by 23.8% in both EC and TMR substrates when compared with the controls. In a similar trend, ETE^L^ and ETE^P^ reduced 24 hour methane production by 16.3% and 12.4%, respectively, in EC substrate, and 16.7% and 21.4%, respectively, in the TMR substrates. Both palm oil only and lard only treatments had no effect on methane production after 24 hours incubation in the EC substrate, but lard only treatment reduced 24 hour methane production in the TMR substrate when compared with the control. Generally, the oil type did not influence the properties of microparticles, as reflected by the lack of significant differences observed between ETE^L^ and ETE^P^ in total gas or methane production. Substrate type and the inclusion of tannin additives did not have any significant interaction effect on methane concentration after 24 h of *in vitro* incubation (*P*> 0.05). However, the inclusion of ATE, ETE^L^, and ETE^P^ incubations only tended to reduce methane concentration in both EC and TMR substrates (*P* = 0.09). Similarly, methane concentration as a result of the inclusion of additives, tends to be lower in the EC substrate compared with the TMR substrate (*P* = 0.06).

**Table 3:** Influence of Acacia tannin extract and lipid-encapsulated Acacia tannin extract (ETE^L^, ETE^P^) on *in vitro* gas production, methane, and fermentation parameters of *Eragrostis curvula* hay and total mixed ration feeds.

^1^Treatment		Total gas (ml/g DM)				Methane (ml/g DM)				^2^Methane (%)	^3^Gas production constants			pH	NH_3_-N
		2 h	4 h	12 h	24 h	2 h	4 h	12 h	24 h		a	b	c		
EC															
Control		11.3	18.2	27.7	47.1^a^	1.04	1.72^a^	2.61	4.92a	10.7	10.3^a^	149.8^b^	0.011	6.94	11.9
Lard		10.7	17.2	28.2	50.1^a^	0.90	1.50^ab^	2.50	4.81^a^	9.61	8.38^a^	155.3^ab^	0.012	6.92	11.7
ETE^L^		10.1	15.6	24.5	43.9^ab^	0.86	1.47^ab^	2.33	4.12^ab^	9.79	7.29^ab^	203.3^a^	0.009	6.76	9.5
Palm oil		8.90	16.0	26.8	47.2^ab^	0.81	1.49^ab^	2.49	4.65^a^	9.86	6.17^b^	231.3^a^	0.010	6.89	12.0
ETE^P^		9.13	15.7	25.3	43.7^ab^	0.80	1.45^ab^	2.37	4.31^ab^	9.86	6.90^b^	200.9^ab^	0.009	6.92	9.0
ATE		6.50	13.0	21.1	37.9^b^	0.58	1.20^b^	1.93	3.75^b^	9.91	5.42^b^	176.3^ab^	0.008	6.87	9.0
TMR															
Control		29.2^a^	50.9^a^	97.6^a^	157.1^a^	1.97	3.79^a^	8.29^a^	16.8^a^	10.7	-42.7^a^	189.6	0.178^a^	6.66	22.0^a^
Lard		27.1^a^	49.0^a^	92.2^a^	152.2^a^	1.89	3.78^a^	8.10^a^	15.7^b^	10.3	-41.3^a^	186.2	0.176^a^	6.67	21.7^a^
ETE^L^		20.9^b^	39.3^b^	79.0^b^	136.1^b^	1.70	3.30^ab^	7.26^b^	14.0^c^	10.3	-32.5^b^	173.7	0.163^b^	6.65	16.8^b^
PalmOil		27.2^a^	49.1^a^	93.3^a^	155.7^b^	2.38	4.24^a^	8.67^a^	16.1^ab^	10.4	-39.6^a^	187.9	0.177^a^	6.59	21.6^a^
ETE^P^		20.8^b^	41.0^b^	78.4^b^	136.4^b^	1.63	2.43^b^	6.09^b^	13.2^c^	9.7	-33.0^b^	172.9	0.162^b^	6.59	16.4^bc^
ATE		19.9^b^	37.8^b^	75.1^b^	127.5^b^	1.55	2.89^b^	6.64^b^	12.8^c^	10.0	-34.6^b^	191.3	0.148^c^	6.78	15.5^c^
SEM		1.36	2.52	5.23	8.55	0.11	0.19	0.45	0.90	0.09	4.71	4.92	0.02	0.03	0.99
^4^*P*-values	S	< .0001	< .0001	< .0001	< .0001	< .0001	< .0001	< .0001	< .0001	0.059	< .0001	0.718	< .0001	< .0001	< .0001
	T	< .0001	< .0001	< .0001	< .0001	0.148	0.003	< .0001	< .0001	0.086	0.001	0.016	< .0001	0.197	< .0001
	S*T	0.004	0.001	< .0001	0.001	0.426	0.027	0.001	< .0001	0.625	< .0001	0.004	0.0001	0.088	< .0001

^1^ETE^L^, lard encapsulated Acacia tannin extract; ETE^P^, palm oil encapsulated Acacia tannin extract; ATE, Acacia tannin extract. SEM, standard error of mean.

^2^Methane, ‘% of methane in the gas sample.

^3^a, gas production from soluble fraction (ml g^-1^ DM); b, gas production from slowly fermentable fraction (mL g^-1^ DM); c, rate of fermentation of fraction ‘b’(mL h^-1^). ^1^24 h methane, mL methane per 100 mL total gas.

^4^*P*-values: S: effect of substrate, T: effect of treatment/additives, S*T: effect of substrate and treatment interaction. For each substrate, mean values within the same column followed by different superscripts differ significantly at *P* ˂ 0.05.

There was a strong interaction effect of substrate type and treatment on the fermentation kinetics (*P*< 0.05). When compared with the control diet, the inclusion of ETE^P^ and ETE^L^ resulted in higher gas production from the rapidly fermentable portion (‘a’ fraction) of the TMR substrate. Similarly, ETE^P^ reduced the ‘a’ fraction of gas production from the EC substrate, when compared with the control, while ETE^L^ did not have any effect (P <0.05). The volume of gas production from the slowly fermentable portion of substrates (‘b’ fraction) showed that in the EC substrate, ETE^L^ and ETE^P^ inclusion increased the ‘b’ fraction when compared with the control while ATE inclusion did not elicit any effect. In contrast, no difference in ‘b’ fraction was observed, across the treatments in the TMR substrate. On the rate of gas production in the TMR substrates, the effect of the encapsulated extract showed that ETE^L^ and ETE^P^ were intermediate between the highest rate observed in the control diet and the lowest rate observed in the crude ATE treatment. In contrast, no difference was observed in the rate of gas production when ETE^L^ and ETE^P^ incubations were compared with the crude ATE or with the control treatment in the EC substrate. The inclusion of extracts did not affect rumen fluid pH after 24 hours incubation (*P*> 0.05), but rumen pH was lower in the TMR substrate compared to the EC substrate (*P* < .0001). The addition of crude ATE, ETE^L^ and ETE^P^ reduced ammonia nitrogen concentration of rumen fluid after 24 h incubation in the TMR substrate but no such effect was observed in the EC substrate. However, the crude ATE reduced rumen ammonia concentration more than that recorded by ETE^L^ when supplemented in the TMR substrates (*P*< 0.0001).

## Discussion

Important considerations in the encapsulation of bioactive products for oral administration are the particle size and EE. The larger particle sizes of microparticles have been related to the higher viscosity of lipid melt compared to lipid solution applicable in the S/O/W emulsion process [32]. For the S/O/W emulsion process, as the ATE/lipid ratio was increased, by increasing the initial weight of ATE dissolved in DCM, there was a decrease in EE and ATE yield percentage (Table 1). As ATE concentration increased at constant volume of the external aqueous phase, mean particle diameter tends to increase. Scanning electron microscopy revealed that spherical particles were produced for all ATE/lipid ratios with indentations on the surface. The generally high concentrations of ATE used in this experiment may be responsible for the indentations, as was observed in a previous report [40]. High concentration of ATE may have resulted in leaching of ATE into the surrounding external aqueous phase, causing the indentation of microparticles surfaces. However, for all ATE concentrations, EE was high, ranging from 62 to 80%. The lower ATE: lipid ratio in batch L-1 may have ensured that a lower amount of core material was dispersed across the phase boundary, thus resulting in higher EE. Another factor that may be responsible for the generally high EE is the nature and amount of surfactant used in both S/O and oil-in-water (O/W) phases. Preliminary trials were conducted to establish the amount of Tween80 and Span80 that would give good emulsion stability. The concentration of the emulsifiers was subsequently kept constant at the levels determined most appropriate. It has been noted that surfactant concentration had a significant impact on the yield and EE of microparticles by contributing to the stabilization of the double emulsion. According to Jiao and Burgess [41], droplet size and stability of emulsions depend on the concentration of both lipophilic and hydrophilic emulsifiers. An increase in the volume of the external aqueous phase also resulted in an increase in EE and particle sizes (Table 1). The concentration of the external aqueous phase (1% Span80) was kept constant for all batches. The increase in microparticle size associated with an increase in the volume of the external aqueous phase can be attributed to a reduction in agitation intensity that occurred with larger volumes during the preparative process, which would result in the formation of larger microparticles. A reduction in mixing efficiency may occur due to an increase in the volume of the external aqueous phase. The present result is consistent with a previous trial [29], in which a decrease in homogenization intensity because of the increased volume of external aqueous phase resulted in reduced microparticle size and EE. However, in the current study, the yield of microparticles increased as the volume of the external aqueous phase increased.

This study envisaged preparing a tannin product that would not dissolve easily under the salivary conditions of the mouth, thus masking the bitter taste of the tannin, but would dissociate in the rumen. While the *in vitro* release protocol is an indication of solubility of the ATE-encapsulating lipid microparticles under the various pH media, it does not fully describe their dissolution under rumen conditions. For example, rumen microbes would expectedly hydrolyse the wall materials to free fatty acid [21,22] thus exposing the core material to rumen fermentation. The *in vitro* release results indicate that the ATE-encapsulated lipid microparticles would not easily be solubilised in saliva during ensilivation, mastication and swallowing, processes that depend on product solubility. An encapsulated fumaric acid preparation had similarly been found to exhibit a sustained release pattern of the core material in rumen fluid [25].

Several researchers [42–44] have reviewed the effect of tanniferous plants or tannin extracts on methanogenesis. This study aimed to develop and evaluate an encapsulated tannin extract for rumen methane mitigation in ruminant animal production. An encapsulated ATE can be useful in decreasing rumen methane production, and bypassing protein from the rumen, among other benefits, without the astringency properties affecting feed intake. With a sustained release system, enteric methane reduction may be enhanced even more effectively. The use of the lipid-encapsulated tannin improved 24 hour gas production slightly compared with the use of the crude ATE. Gas production is an indication of dry matter fermentation, particularly the carbohydrate components of the feed [36,45]. This showed that the reduction in gas production often associated with inclusion of tannin was slightly curbed as a result of the encapsulation of tannin, which makes them less available per time. However, the emission intensity, as judged in terms of mL of methane produced per 100 mL of total gas showed that only slight reduction in methane intensity could be associated with the inclusion of the tannin additives, either ATE, ETE^L^ or ETE^P^. The effect of condensed tannins observed in the current study appeared to be associated more with reduced fermentation of dry matter than reduced activity of methanogens. The anti-methanogenic activities of condensed tannins have been related to a combination of direct toxicity on methanogenic archaea, reduced fibre degradation or reduced OM digestibility [44].

Substrate type is known to influence microbial population, and tannins have been noted to exhibit varying inhibitory effects on various rumen microbes [46]. In this study, substrate type only tended to influence the antimethanogenic activity of the tannin extracts as seen from the methane concentrations. In a high crude protein diet such as the TMR, it appeared that the antimethanogenic effects of tannins became less obvious compared with lower crude protein diets such as the Eragrostis hay substrate. This interaction between diet type and methane production, following tannin inclusion had been reported previously [47]. A gradual release of tannin from the microparticles upon dissociation of the wall materials may be responsible for higher gas production recorded in the ETE^L^ and ETE^P^ treatments when compared with the crude ATE treatment. Inclusion of lard or palm oil only did not have any effect on gas production.

The results of reduced gas production associated with tannin reported in this study agrees with those of various researchers on the effect of condensed tannin on *in vitro* gas production, as was observed for plants with high CT content [47] or incubations with tannin extracts [42,46]. Lipid-encapsulated tannin (ETE^P^ and ETE^L^) resulted in higher gas production compared with the crude ATE treatment, despite containing equivalent amounts of the active ingredient. This may be related to the reduction in initial solubilisation of substrate and tannin extract in the mixed rumen fluid or the reduction in the rate of attachment between the mixed microbial population and the incubation ingredients as can be seen in slightly higher ‘a’ fraction recorded in ETE^L^ compared with ATE, in the Eragrotis hay substrate. Similar to the result in the current study, Dentinho et al. [48] observed a significant reduction in initial solubilisation and subsequent degradation of soybean meal as a result of tannin extract inclusion. Negative values of ‘a’ fraction of substrates have been reported in previous trials [49,50] when using gas production mathematical models and can be attributed to a deviation from the exponential course of fermentation or prolonged lag phase for microbial attachment.

Although the rate of Eragrostis hay fermentation was not different in ETE^L^ and ETE^P^ compared with the ATE treatment, the fermentation rate was significantly higher in the TMR substrate up to 24 hours incubation as a result of the encapsulated ATE inclusion, rather than the crude ATE. For the degradation parameters, “b” represents components that are progressively but slowly fermented, while ‘c’ represents the rate of degradation of ‘b’. Tannin extracts have been reported to slow the rate of dry matter digestion *in vitro* [42], but with encapsulation, a higher degradation rate can be achieved. The effects of condensed tannin on *in vitro* fermentation and nutrient digestibility depend largely on the formation of complexes with proteins and, to a lesser extent, fibre, as well as their effects on the mixed microbial population [47]. In the TMR substrate, the higher ammonia concentration observed in the lipid-encapsulated ATE treatments compared with the crude ATE treatment is an indication that encapsulation of ATE can influence the activity of rumen fermentation positively, depending on the diet characteristics. The encapsulation of tannin did show some promise in improving gas production compared to the crude ATE, while still exhibiting typical tannin activities such as antimethanogenesis.

## Conclusion

The present study showed that lard or palm oil could be used to encapsulate a tannin extract. The lipid-encapsulated ATE microparticles prepared using the S/O/W process exhibited good morphological characteristics, had high encapsulation efficiencies, and displayed sustained release of the tannin extract over time. The lipid-encapsulated microparticles also reduced total gas and methane production *in vitro* and therefore, could be utilized to modulate rumen fermentation positively.

## Supporting information

S1 Table Raw Excel data on lipid-encapsulated Acacia tannin microparticles. (XLSX)
